# Promoted Neuronal Differentiation after Activation of Alpha4/Beta2 Nicotinic Acetylcholine Receptors in Undifferentiated Neural Progenitors

**DOI:** 10.1371/journal.pone.0046177

**Published:** 2012-10-04

**Authors:** Takeshi Takarada, Noritaka Nakamichi, Seiya Kitajima, Ryo Fukumori, Ryota Nakazato, Nguyen Quynh Le, Yeong-Hun Kim, Koichi Fujikawa, Miki Kou, Yukio Yoneda

**Affiliations:** Laboratory of Molecular Pharmacology, Division of Pharmaceutical Sciences, Kanazawa University Graduate School of Natural Science and Technology, Kanazawa, Ishikawa, Japan; University of Iowa, United States of America

## Abstract

**Background:**

Neural progenitor is a generic term used for undifferentiated cell populations of neural stem, neuronal progenitor and glial progenitor cells with abilities for proliferation and differentiation. We have shown functional expression of ionotropic N-methyl-D-aspartate (NMDA) and gamma-aminobutyrate type-A receptors endowed to positively and negatively regulate subsequent neuronal differentiation in undifferentiated neural progenitors, respectively. In this study, we attempted to evaluate the possible functional expression of nicotinic acetylcholine receptor (nAChR) by undifferentiated neural progenitors prepared from neocortex of embryonic rodent brains.

**Methodology/Principal Findings:**

Reverse transcription polymerase chain reaction analysis revealed mRNA expression of particular nAChR subunits in undifferentiated rat and mouse progenitors prepared before and after the culture with epidermal growth factor under floating conditions. Sustained exposure to nicotine significantly inhibited the formation of neurospheres composed of clustered proliferating cells and 3-[4,5-dimethylthiazol-2-yl]-2,5-diphenyltetrazolium bromide reduction activity at a concentration range of 1 µM to 1 mM without affecting cell survival. In these rodent progenitors previously exposed to nicotine, marked promotion was invariably seen for subsequent differentiation into cells immunoreactive for a neuronal marker protein following the culture of dispersed cells under adherent conditions. Both effects of nicotine were significantly prevented by the heteromeric α4β2 nAChR subtype antagonists dihydro-β-erythroidine and 4-(5-ethoxy-3-pyridinyl)-*N*-methyl-(3*E*)-3-buten-1-amine, but not by the homomeric α7 nAChR subtype antagonist methyllycaconitine, in murine progenitors. Sustained exposure to nicotine preferentially increased the expression of *Math1* among different basic helix-loop-helix proneural genes examined. In undifferentiated progenitors from embryonic mice defective of NMDA receptor subunit-1, nicotine was still effective in significantly inhibiting the proliferation.

**Conclusions/Significance:**

Functional α4β2 nAChR subtype would be constitutively expressed to play a role in the mechanism underlying the determination of proliferation and subsequent differentiation fate into a neuronal lineage in association with preferential promotion of Math1 expression in undifferentiated neural progenitors of developing rodent neocortex independently of NMDA receptor activation.

## Introduction

Neurodegenerative disorders are regarded as progressive loss of neurons in specific brain areas, resulting in significant cognitive and motor disability. These include Alzheimer's, Parkinson's and Huntington's diseases, and amyotrophic lateral sclerosis. In recent years, clinical interests are increasing in regeneration therapies for those patients with neuronal loss using endogenous neural stem cells. Neural stem cells are primitive cells with self-renewal capacity and multi-potentiality to generate different neural lineages including neurons, astroglia, and oligodendroglia. Cells with these characteristics are abundant throughout the brain during embryonic and postnatal development [1], [2], while in the adult brain progenitor cells are highly localized in the dentate gyrus (DG) of hippocampus as well as the subventricular zone (SVZ) [3]–[7]. These neural progenitors undergo cellular proliferation, commitment, and differentiation into neurons and glia *in vitro* [8], suggesting that these cells are indeed derived from multi-potential neural stem cells [9]. The fact that on transplantation of neural stem cells into brains, they develop into mature neurons or glia with morphological and biochemical features similar to those of neighboring cells, gives rise to an idea that cellular commitment and/or differentiation is at least in part under the influence by the microenvironments around the stem cells *in vivo* [10].

Emerging evidence that endogenous factors regulate self-renewal capacity and multi-potentiality of neural progenitors expressed in the developing and matured brains is available in the literature. Several molecules, such as growth factors and neurotransmitters, have been implicated in the extrinsic regulation of cell proliferation in the developing telencephalon. For example, basic fibroblast growth factor (bFGF) prolongs the proliferation period of progenitor cells with a concomitant increase in the number of neurons in rat neocortex when either added to cultured cells *in vitro* [11] or microinjected into embryonic rat brains *in vivo* [12]. The neurotransmitters glutamate and gamma-aminobutyric acid (GABA) reduce the number of proliferating cells in dissociated or organotypic cultures of rat neocortex [13]. We have also shown that the systemic administration of the glutamate analog, N-methyl-D-aspartate (NMDA), decreases the cellular proliferation in the DG of adult murine hippocampus in a manner sensitive to an NMDA receptor (NMDAR) antagonist [14]. The systemic administration of NMDA also markedly reduces expression of the neural progenitor marker protein, nestin, and proliferating cell nuclear antigen in the DG, without significantly affecting their expression in the SVZ [14]. Moreover, sustained activation of NMDAR not only decreases the size of neurospheres formed by clustered cells, but also facilitates the neuronal commitment induced by all-*trans* retinoic acid (ATRA), in cultured neural progenitor cells isolated from adult mouse hippocampus [15].

Nicotinic acetylcholine receptors (nAChRs) are a member of the superfamily of ligand-gated ion channels found throughout the mammalian central nervous system (CNS), with unique localization in pre-, post-, and extrasynaptic membranes [16]. Large numbers of distinct subtypes of nAChRs are found with different subunit compositions in the CNS. In mammals, nAChRs are composed of an assembly of five receptor subunits, which may be homomeric assemblies of an alpha (α7 or α9) subunit or alternatively some combinations between alpha (α2–α6) and beta (β2–β4) subunits [17], [18]. The most abundant nAChR subtype is the α4β2 heteromer in the brain, a homomeric α7 assembly being the other major subtype, whereas other subunits such as α2, α3, α5, α6, β2, and β4 would be responsible for heteromeric orchestrations [19]. Activation of nAChRs could often increase intracellular free Ca^2+^ levels through the direct passage of extracellular Ca^2+^ across the receptor channels in neurons, in addition to Na^+^ and K^+^ [20], [21]. Among these nAChR subtypes in the brain, the homomeric α7 subtype would exhibit higher permeability to Ca^2+^ than the other ligand-gated ion channels permeable to this divalent cation, NMDAR [22], [23].

In this study, we have attempted to evaluate the possible importance of signal inputs mediated by different nAChR subtypes in the mechanism related to self-replication and multi-potentiality in neural progenitor cells isolated from fetal rodent neocortex *in vitro*.

## Materials and Methods

### Materials

Propidium iodide (PI), Hoechst33342, 3-[4,5-dimethylthiazol-2-yl]-2,5-diphenyltetrazolium bromide (MTT), antibodies against microtubule-associated protein-2 (MAP2) (monoclonal mouse IgG1 isotype) and glial fibrillary acidic protein (GFAP) (rabbit IgG fraction of antiserum) were purchased from Sigma Chemical Company (St Louis, MO, USA). An anti-mouse IgG antibody conjugated with Alexa488, an anti-rabbit IgG antibody conjugated with Alexa594, anti-goat IgG antibody conjugated with Alexa594 were obtained from Invitrogen (San Diego, CA, USA). Dulbecco's modified Eagle's medium: nutrient mixture F-12 (DMEM/F-12) was purchased from GIBCO BRL (Gaithersburg, MD, USA). Epidermal growth factor (EGF) was provided by Biomedical Technologies (Stoughton, MA, USA). ISOGEN was purchased from Nippon Gene Co. (Tokyo, Japan). rTaq DNA polymerase was obtained from Takara Bio Inc. (Otsu, Japan). Dihydro-β-erythroidine (DHβE), methyllycaconitine (MLA) and 4-(5-ethoxy-3-pyridinyl)-*N*-methyl-(3*E*)-3-buten-1-amine (TC2559) were purchased from Tocris (Bristol, UK). Fluo-3 acetoxymethyl ester was obtained from Molecular Probes (Eugene, OR, USA). Antibodies against α4 and β2 nAChR subunits were provided by Santa Cruz Biotechnology (Santa Cruz, CA, USA).

### Preparation of neural progenitor cells

This study was carried out in strict accordance with the recommendations in the Guide for the Care and Use of Laboratory Animals of the Japanese Society for Pharmacology. The protocol was approved by the Committee on the Ethics of Animal Experiments of the University of Kanazawa (Permit Number: 101605) with an effort to minimize the number of animals used and their suffering. Neocortex was isolated from 15.5-day-old embryonic Std-ddY mice or 18-day-old embryonic Wistar rats, cleared of meninges and incubated in Ca^2+^, Mg^2+^-free phosphate-buffered saline (PBS; 137 mM NaCl, 8.1 mM Na_2_HPO_4_, 2.68 mM KCl, and 1.47 mM KH_2_PO_4_) containing 50 mM glucose, 100 U/ml penicillin, 100 µg/ml streptomycin, and 0.25 mM N-acetyl-L-cysteine for 20 min at 37°C with gentle shaking. The tissue sediments were then mechanically triturated through Pasteur pipettes with a fire-narrowed tip in an enzyme cocktail solution containing 2.5 U/ml papain, 250 U/ml Dnase, and 1 U/ml neutral protease in PBS at 37°C for 30 min as described previously [14], [15]. Cell suspensions were washed three times with DMEM/F-12 supplemented with 10% fetal bovine serum (FBS) and then mixed with an equal volume of Percoll solution made by mixing nine parts Percoll with one part 10×PBS. Cell suspensions were centrifuged at 20,000 g for 30 min at 18°C, which resulted in two different cell layers based on the Percoll density gradient, followed by gentle aspiration of resultant upper and lower cell layers [14]. Cell fractions collected in the lower layer were harvested and washed three times with DMEM/F-12 containing 10% FBS [15]. Cell suspensions (15,000 cells) were seeded with 0.5 ml aliquots per well in culture plates (1.9 cm^2^, 24 wells; Nalge Nunc International, Roskilde, Denmark). Cells were cultured for a period of up to 10 days for mouse progenitors or 12 days for rat progenitors in the absence of FBS in DMEM/F-12 growth medium containing 0.6% glucose, 15 mM sodium bicarbonate, 250 mM N-acetyl-L-cysteine, 10 ng/ml EGF, 20 nM progesterone, 30 nM sodium selenite, 60 nM putrescine, 25 µg/ml insulin, and 100 µg/ml apo-transferrin. Cells were cultured at 37°C under 5% CO_2_ in a humidified CO_2_ incubator under floating conditions with a half medium change every 2 days.

**Figure 1:**
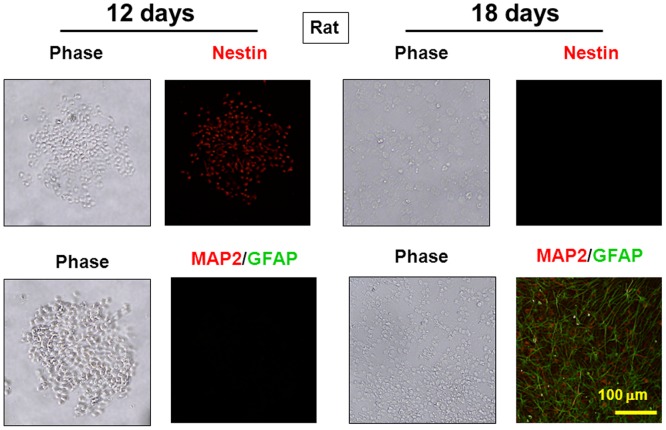
Validation of procedures for neural progenitor isolation. Neocortex was dissected from fetal rat brains, followed by preparation of lower cell layer fractions and subsequent culture with EGF for 12 days under floating conditions. Cells were then dispersed and cultured without EGF for 6 days under adherent conditions. Cells were fixed for immunocytochemistry analysis on the progenitor marker nestin, the neuronal marker lMAP2 and astroglial marker GFAP.

In rat neurospheres, for example, cells were highly immunoreactive for the progenitor marker nestin, but not for the neuronal marker MAP2 or the astroglial marker GFAP on Day 12 (Fig. 1, left pictures). Cells were then dispersed and seeded in a 24-well plate previously coated with poly-L-lysine toward further culture after the removal of EGF for the spontaneous differentiation for an additional 6 days. Removal of EGF led to an appearance of cells with marked expression of either MAP2 or GFAP, with no cells immunoreactive for nestin on Day 18 (Fig. 1, right pictures).

### Reverse transcription polymerase chain reaction (RT-PCR)

Total RNA was extracted from cultured neural progenitor cells using the standard ISOGEN procedure and then subjected to the synthesis of cDNA. The individual cDNA species were amplified in a reaction mixture containing a cDNA aliquot, PCR buffer, dNTPs, relevant sense and antisense primers (Table 1), and rTaq DNA polymerase. Reactions were initiated by incubating at 95°C for 10 min and PCR (denaturation at 95°C for 1 min, annealing at 62°C for 1 min, and extension at 72°C for 1 min) was performed with a final extension at 72°C for 5 min. In preliminary experiments, a clearly linear correlation was optimized with each primer set, respectively. Quantitative analysis was done at the cycle number with high linearity between mRNA expression and cDNA production using primers for the housekeeping gene glyceraldehyde-3-phosphate dehydrogenase (GAPDH). PCR products were quantified by using densitograph, followed by calculation of ratios of expression of mRNA for each gene over that for GAPDH.

**Table 1:** Primers used in this study.

Genes	Upstream (5′-3′)	Downstream (5′-3′)
**Mouse nAChR**		
α2	TCTACCCCGACGTCACCTAC	ACACCCATGGAAGAGTCTGG
α3	TGGGGATTTCCAAGTGGA	CATGACCCTGGGGAGAAGGTT
α4	CAATGTACACCACCGCTCAC	TGGTCTGACACTGGAAGCTG
α5	CCTCTGCTGCAAAACATGAA	CGCTCATGATTTCCCATTCT
α6	TCCCTGAAGTTTGGTTCCTG	CTCCTGCCTCCTTTGTCTTG
α7	GCACCTCATGCATGGTACAC	ATCCAGAGTGGGCAATGAC
α9	CCTTGCGTCCTCATATCGTT	CCCTGGAAGTTTGCCATAAA
β2	GGGAAGATTATCGCCTCACA	GCCAGCAGCACAGAAATACA
β3	GCTCAGTGGCTGAACATGAA	TCTTCTGTTGCCCTTCATCC
β4	TCTGGTTGCCTGACATCGTG	GGGTTCACAAGTACATGGA
**Rat nAChR**		
α2	TGCCCAGGTGGCTGATGATGAACC	GCTTTCTGTATTTGAGGTGACAGC
α3	AACCTGCTCCCCAGGGTCATGTTT	CACTTTGGATGGCTTCTTTGATTT
α4	GTCAAAGACAACTGCCGGAGACTT	TGATGAGCATTGGAGCCCCACTGC
α5	GTGGATTTAGTGAGCAGTCATGCA	TTTGGGGGGAGTTTTAAATAGTCT
α6	CAGGTCTTCCCCTCGATTCTGATG	CATTGTGGCTTTTCATGTTTTCTG
α7	AACTGGTGTGCATGGTTTCTGCGC	AGATCTTGGCCAGGTCGGGGTCCC
α9	ATCCTGAAGTACATGTCCAGGATC	TGGCCTTGTGGTCCTTGAGGCACT
β2	ACGGTGTTCCTGCTGCTCATC	CACACTCTGGTCATCATCCTC
β3	GAAGATGTGGATACATCGTTTCCA	GAGCAGAGGGAGTAGTTCAGGAAC
β4	ATGAAGCGTCCCGGTCTTGAAGTC	GGTCATCGCTCTCCAGATGCTGGG
**bHLH transcriptional factor**		
Mash1	AACAAACCAGACAGCCAACC	AAAGGCTGTCCGAGAACTGA
Math1	GCTTCCTCTGGGGGTTACTC	ACAACGATCACCACAGACCA
Math3	TCTTCGACTGGCAAGGAACT	ACTAATGCTCAGGGGTGGTG
NeuroD1	CAAAGCCACGGATCAATCTT	CCCGGGAATAGTGAAACTGA
Ngn3	TGCAGCCACATCAAACTCTC	GCAGAAGAAGGCAGATCACC
Hes1	GCCAATTTGCCTTTCTCATC	AGGCGCAATCCAATATGAAC
Hes5	ATGCTCAGTCCCAAGGAGAA	CGCTGGAAGTGGTAAAGCAG
Others		
CyclinD1	AGTGCGTGCAGAAGGAGATT	CACAACTTCTCGGCAGTCAA
Gapdh	ACCACAGTCCATGCCATCAC	TCCACCACCCTGTTGCTGTA

### Cell viability

Cells were cultured in either the presence or absence of drugs, followed by re-plating on dishes previously coated with poly-L-lysine for the further culture for 2 h and subsequent fixation with 4% paraformaldehyde for double staining for DNA with 10 µg/ml Hoechst 33342 and 5 µg/ml PI. The number of cells stained with these dyes were individually counted for calculation of the percentages of dead cells stained with the membrane-impermeable dye PI over the total cells stained with the membrane-permeable dye Hoechst33342.

Cell viability was also quantified by MTT reduction assays [24] with minor modifications [25]. In brief, culture medium was replaced with PBS containing 0.5 mg/ml MTT and incubated for 1 h at 37°C. Cells were then solubilized in a lysis solution containing 99.5% isopropanol and 0.04 M HCl. The amount of MTT formazan product was determined by measuring the absorbance at 550 nm on a microplate reader. Relative values were calculated by percentages over control values obtained in a parallel control experimental group. The activity of lactate dehydrogenase (LDH) released into the culture medium was determined by the enzyme-coupled color development method as descried previously [26] with minor modifications.

### Neurosphere differentiation

Cells were cultured for 10 to 12 days in the presence of 10 ng/ml EGF under floating conditions as described above, and then medium was replaced with DMEM/F-12 medium containing 1% FBS, 15 mM sodium bicarbonate, 250 nM N-acetyl-L-cysteine, 100 U/ml penicillin, and 100 µg/ml streptomycin 10 to 12 days after plating. These cells were suspended at a density of 30,000 cells/ml, followed by seeding in four-well plates previously coated with poly-L-lysine and subsequent culture for an additional 4 to 6 days in the absence of EGF under adherent culture conditions to initiate spontaneous differentiation. Cells were also cultured in the presence of all-*trans* retinoic acid (ATRA) at 100 ng/ml for facilitation of the neuronal differentiation or ciliary neurotrophic factor (CNTF) at 20 ng/ml for facilitation of the astroglial differentiation for an additional 4 to 6 days as needed.

### Immunocytochemistry

Cells were washed with PBS, followed by fixation with 4% paraformaldehyde for 20 min at 4°C and subsequent blocking with 10% normal horse serum or goat serum in PBS containing 0.1% Triton X [27]. Cells were then reacted with antibodies appropriately diluted against MAP2 (1∶500) and GFAP (1∶500) overnight at 4°C. Finally, cells were reacted with the corresponding secondary antibody (1∶600), anti-mouse IgG antibody conjugated with Alexa488, or anti-rabbit IgG antibody conjugated with Alexa594, and observed under a confocal laser-scanning microscope (LSM 510; Carl Zeiss, Jena, Germany). Quantification was performed by counting the number of cells immunoreactive for either MAP2 or GFAP on double immunocytochemistry analysis, followed by calculation of the individual percentages over the number of total cells stained with Hoechst33342. Because of the resolution limitation of fluorescence microscopy, cells with slight colors were all counted as cells immunoreactive for either antibody in a blinded fashion.

The lower cell layer obtained after percoll centrifugation was also cultured in DMEM/F-12 containing 10 ng/ml EGF for 6 days, followed by plating on a dish previously coated with poly-L-lysine for slight adhesion during further culture for an additional 2 h and subsequent washing in PBS for fixation with 4% PA. Cells were then subjected to immunocytochemical detection of α4 and β2 subunits of nAChR as described above.

### Measurement of intracellular free Ca^2+^ levels

Floating neurospheres were harvested on Day 10, followed by medium replacement with recording medium composed of 10 mM HEPES buffer (pH 7.4) containing 129 mM NaCl, 4 mM KCl, 1 mM MgCl_2_, 2 mM CaCl_2_ and 4.2 mM glucose, and subsequent incubation at 37°C for an additional 1 h in recording medium containing 30 nM Pluronic F-127 and 3 µM fluo-3 acetoxymethyl ester that is a membrane-permeable form of the Ca^2+^-sensitive dye. Cultures were then washed with recording medium twice, followed by settlement for at least 1 h in the recording medium and subsequent placement of the four-well dish under a confocal laser scanning microscope as described previously [25]. Medium was changed once more, followed by cumulative addition of nicotine at concentrations up to 1 mM and subsequent determination of the fluorescence intensity every 2 min. The fluorescence intensity was normalized after the addition of the Ca^2+^ ionophore A23187 at 10 µM. Drugs were prepared in recording medium immediately before each use. Fluorescence images labeled with fluo-3 were collected using an excitation wavelength of 488 nm. The parameters of illumination and detection were digitally controlled to keep the same settings throughout the experiments.

### Western blotting analysis

Cultured cells were washed with PBS for harvest, followed by lysis in 10 mM PIPES buffer (pH 6.8) containing 100 mM NaCl, 300 mM sucrose, 3 mM MgCl_2_, 1 mM EDTA and 0.5% Triton X-100. Aliquots of extracts were added at a volume ratio of 4∶1 to 10 mM Tris-HCl buffer (pH 6.8) containing 10% glycerol, 2% sodium dodecylsulfate, 0.01% bromophenol blue and 5% 2-mercaptoethanol, followed by mixing and boiling at 100°C for 5 min. An aliquot of 10 µg proteins was loaded on a 7.5% polyacrylamide gel for electrophoresis at a constant current of 15 mA/plate for 2 h at room temperature and subsequent blotting to a polyvinylidene fluoride membrane previously treated with 100% methanol. After blocking by 5% skimmed milk dissolved in 20 mM Tris-HCl buffer (pH 7.5) containing 137 mM NaCl and 0.05% Tween 20, the membrane was reacted with an antibody against either α4 or β2 subunit diluted with buffer containing 1% skimmed milk, followed by reaction with an anti-goat or anti-rabbit IgG antibody conjugated with peroxidase. Proteins reactive with those antibodies were detected with the aid of ECL™ detection reagents through exposure to X-ray films [15].

### Mice defective of NMDAR1 subunit

NMDAR1-null mice were kindly donated by Dr. Thomas Curran (Children's Hospital of Philadelphia, Philadelphia, PA, USA) through Dr. Shigeo Okabe (University of Tokyo, Tokyo, Japan). All experimental procedures were approved by the Institutional Animal Care and Use Committee and conform to the relevant regulatory standards. Neocortex was dissected from 15.5-day-old embryonic mice for subsequent preparation of progenitors as described above. Genotyping was performed by PCR analysis of genomic DNA with each well containing cells from one embryo.

### Data analysis

Quantitative data are expressed as the mean ± SEM, and the statistical significance was determined by the two-tailed Student's t-test or the one-way ANOVA with Bonferroni/Dunnett post hoc test with the level of significance set at p<0.05.

## Results

### Expression of nAChR subunits in rat progenitors

**Figure 2:**
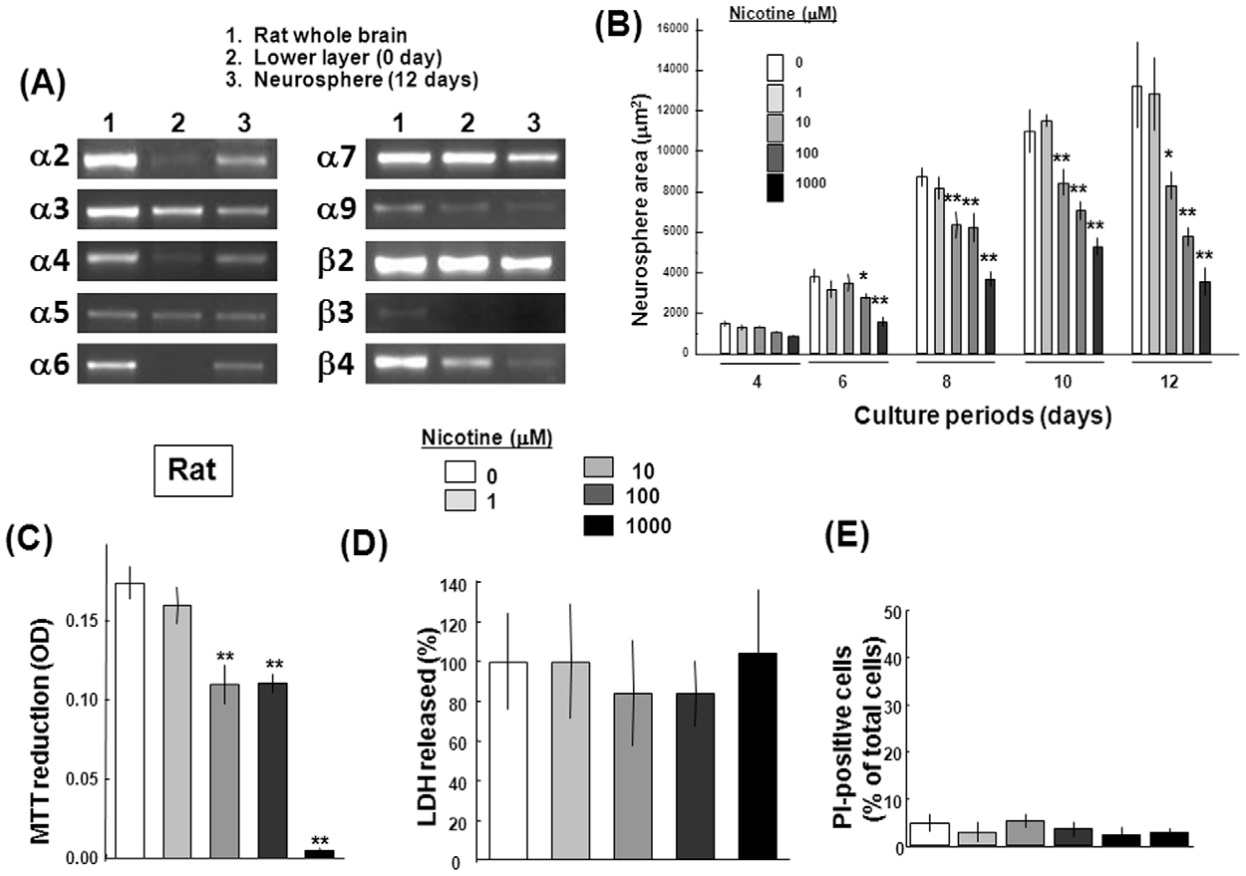
Expression of nAChR subunits in undifferentiated rat neural progenitors. Neocortex was dissected from fetal rat brains, followed by preparation of the lower cell layer fractions. (A) Total RNA was extracted from cells before or after the culture in the presence of EGF for 12 consecutive days for RT-PCR analysis. Adult rat whole brain was used as a positive control. Typical pictures are shown with similar results in three independent sets of experiments. Undifferentiated progenitors were cultured with EGF in the presence of nicotine at different concentrations for determination of (B) the total size during culture, (C) MTT reduction, (D) LDH release and (E) the ratio of PI-positive cells cultured for 12 days. *P<0.05, **P<0.01, significantly different from each control value obtained in cells not exposed to nicotine.

An RT-PCR analysis revealed mRNA expression of several nAChR subunits in lower cell layer fractions prepared from embryonic rat neocortex. These included mRNA for α2, α3, α4, α5, α7, α9, β2 and β4 subunits, but not for α6 and β3 subunits (Fig. 2A). In neurospheres cultured with EGF for 12 days, similar mRNA expression profiles were seen for all the subunits described above. The size of neurospheres was drastically increased in proportion to increasing periods of culture with EGF from 4 to 12 days, while sustained exposure to nicotine resulted in a significant decrease in the size of neurospheres formed from clustered proliferating cells at a concentration range of 10 µM to 1 mM (Fig. 2B). Similarly significant inhibition was found in MTT reduction in cells cultured with nicotine for 12 days (Fig. 2C). Although 1 mM nicotine almost completely abolished MTT reduction in cells cultured for 12 days, nicotine did not significantly affect LDH release (Fig. 2D) or the ratio of PI-positive cells over Hoechst33342-positive cells (Fig. 2E) on Day 12 even at the highest concentration used.

**Figure 3:**
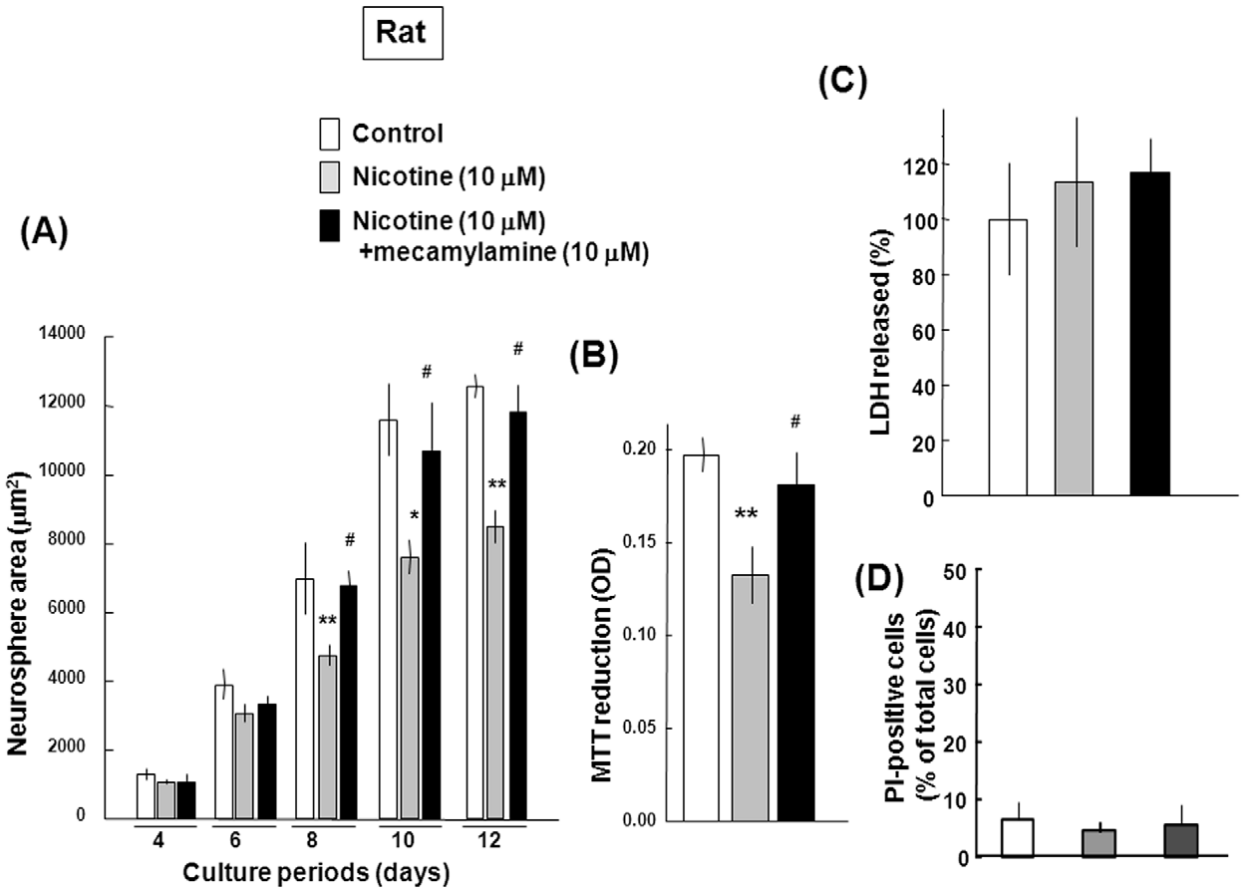
Effects of mecamylamine on cellular viability in rat progenitors. Undifferentiated progenitors were cultured with EGF in either the presence or absence of 10 µM nicotine and 10 µM mecamylamine for a period up to 12 days for determination of (A) neurosphere size, (B) MTT reduction, (C) LDH release and (BD) the ratio of PI-positive cells. *P<0.05, **P<0.01, significantly different from each control value obtained in cells not exposed to nicotine. ^#^P<0.05, significantly different from the value obtained in cells exposed to nicotine alone.

The general nAChR antagonist mecamylamine was effective in significantly preventing the inhibition by 10 µM nicotine of the neurosphere size seen during the periods from 8 to 12 days (Fig. 3A) and of MTT reduction determined on Day 12 (Fig. 3B), but failed to significantly affect LDH release (Fig. 3C) and the ratio of PI-positive cells over Hoechst33342-positive cells (Fig. 3D).

### Differentiation of rat neurospheres

**Figure 4:**
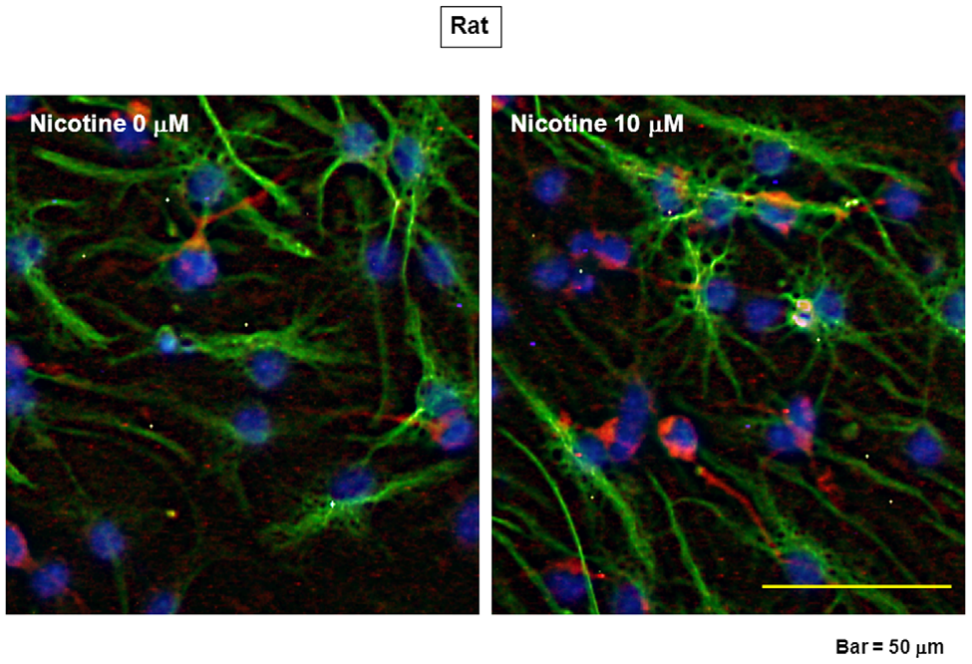
Differentiation of rat progenitors. Cells were dispersed after the culture with EGF in either the presence or absence of 10 µM nicotine for 12 days, followed by further culture for an additional 6 days. Cells were then fixed for double immunocytochemical detection of both MAP2 and GFAP together with Hoechst33342 staining.

**Figure 5:**
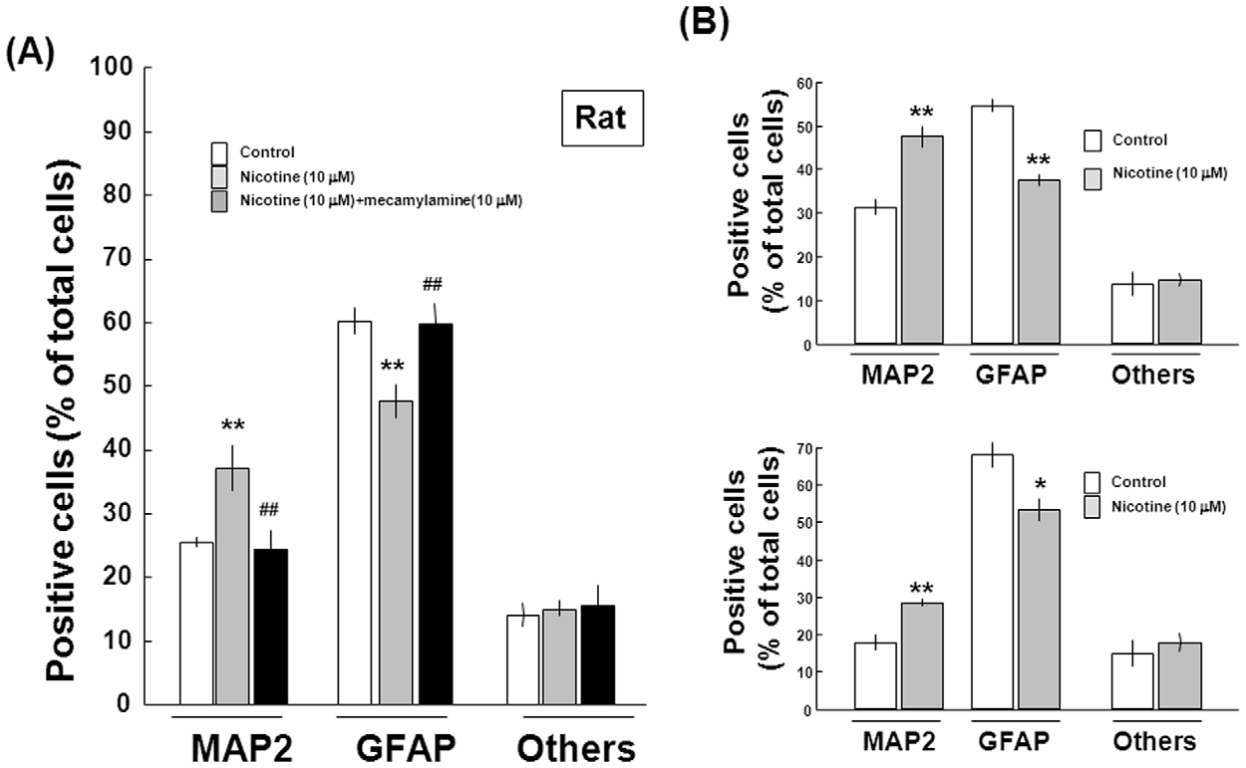
Effects of nicotine on differentiation of rat progenitors. Cells were dispersed after the culture with EGF in either the presence or absence of 10 µM nicotine for 12 days, followed by further culture in (A) the absence and (B) presence of differentiation inducers such as ATRA and CNTF for an additional 6 days. Cells were then fixed for double immunocytochemical detection of both MAP2 and GFAP, followed by counting of the number of individual immunoreactive cells. *P<0.05, **P<0.01, significantly different from each control value obtained in cells not exposed to nicotine. ^##^P<0.01, significantly different from the value obtained in cells exposed to nicotine alone.

Undifferentiated rat progenitors were dispersed after sustained exposure to 10 µM nicotine, followed by further culture in the absence of EGF for an additional 6 days under adherent conditions and subsequent double immunocytochemistry analysis along with Hoechst33342 staining. Removal of EGF led to the appearance of numerous cells immunoreactive for either MAP2 or GFAP on Day 18, while prior culture with nicotine increased the number of cells immunoreactive for MAP2 (Fig. 4). Quantification by counting the number of cells clearly revealed that prior exposure to 10 µM nicotine significantly increased the number of cells immunoreactive for MAP2 with a concomitant decrease in that for GFAP in a manner sensitive to the prevention by 10 µM mecamylamine (Fig. 5A). Prior exposure to nicotine also significantly increased the number of MAP2-positive cells along with decreased GFAP-positive cells, which occurred irrespective of the presence of the neuronal inducer ATRA and the astroglial inducer CNTF during the culture for 6 days, respectively (Fig. 5B).

### Expression of nAChR subunits in mouse progenitors

**Figure 6:**
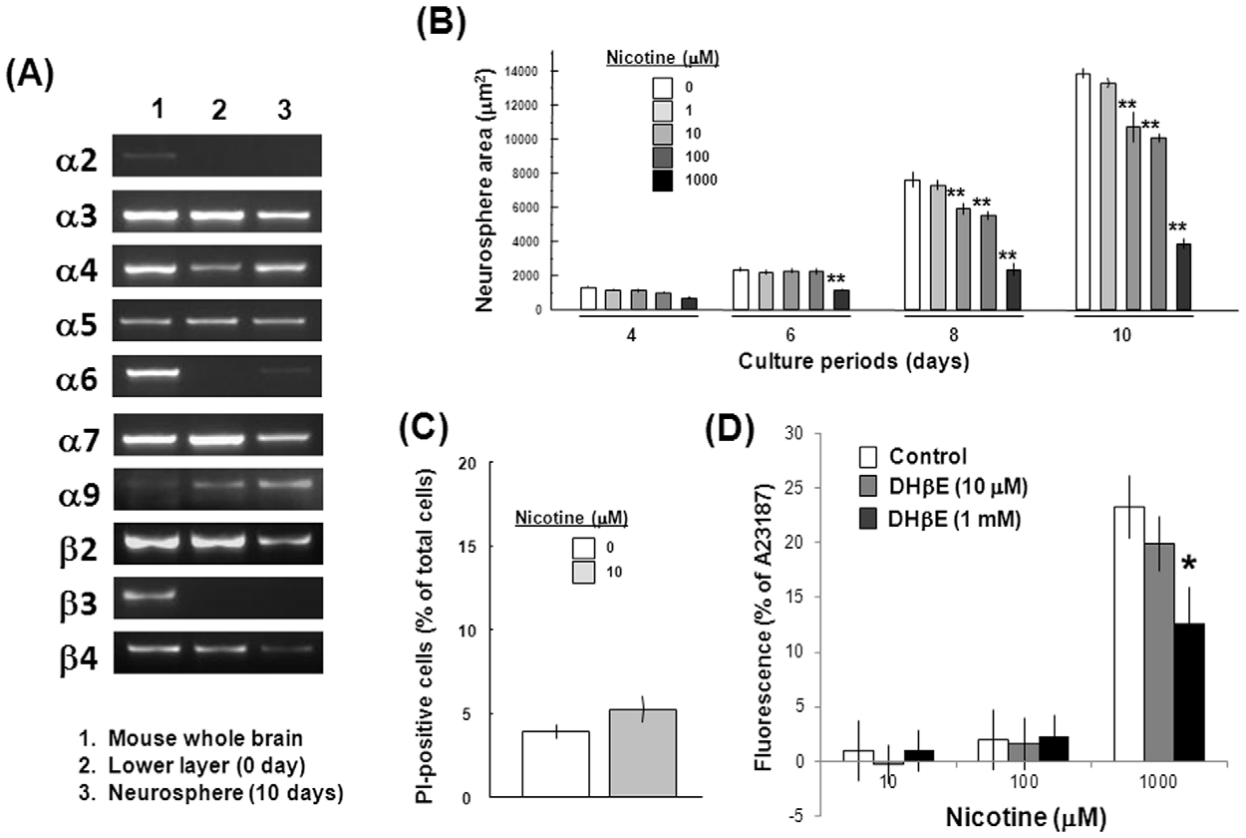
Expression of nAChR subunits in undifferentiated mouse neural progenitors. Neocortex was dissected from fetal mouse brains, followed by preparation of the lower cell layer fractions. (A) Total RNA was extracted from cells before or after the culture with EGF for 10 consecutive days. Adult mouse whole brain was used as a positive control. Typical pictures are shown with similar results in three independent sets of experiments. Undifferentiated progenitors were cultured with EGF in the presence of nicotine at different concentrations for 10 days for determination of (B) the total size during culture and (C) the ratio of PI-positive cells cultured for 10 days in neurospheres. **P<0.01, significantly different from each control value obtained in cells not exposed to nicotine. (D) Undifferentiated progenitors cultured with EGF for 10 days were loaded with fluo-3, followed by cumulative addition of nicotine at a concentration range of 10 µM to 1 mM in either the presence or absence of DHβE at 10 µM and 1 mM and subsequent determination of the fluorescent intensity every 2 min. The individual fluorescence was normalized with the maximal fluorescence intensity obtained after addition of the Ca^2+^ ionophore A23187 at 10 µM.

On RT-PCR analysis of embryonic mouse neocortical lower cell layer fractions before culture, mRNA expression was seen for α3, α4, α5, α7, α9, β2 and β4 subunits of nAChR, but not for α2, α6 and β3 subunits (Fig. 6A), in contrast to those from embryonic rat neocortex. In neurospheres cultured with EGF for 10 days, mRNA expression profiles were not significantly different from those seen in cells before culture. The size of neurospheres was again drastically increased in proportion to increasing culture periods from 4 to 10 days, whereas nicotine significantly decreased the total area of neurospheres at concentrations of 10 µM to 1 mM (Fig. 6B). A negligible number of cells were stained with the membrane-impermeable dye PI for DNA in murine neurospheres exposed to 10 µM nicotine for 10 days, along with most cells being stained with the membrane-permeable dye Hoechst33342 (Fig. 6C). Activation of α7 nAChR subtype is shown to increase intracellular free Ca^2+^ levels due to direct and indirect pathways in PC12 cells [28]. Neurospheres cultured with EGF for 10 days were thus loaded with the Ca^2+^-sensitive fluorescent dye fluo-3, followed by exposure to nicotine and subsequent determination of fluorescence images. Nicotine was effective in drastically increasing the fluorescence intensity during the exposure for 5 min at the highest concentration used of 1 mM in a manner sensitive to the prevention by DHβE at 1 mM (Fig. 6D).

**Figure 7:**
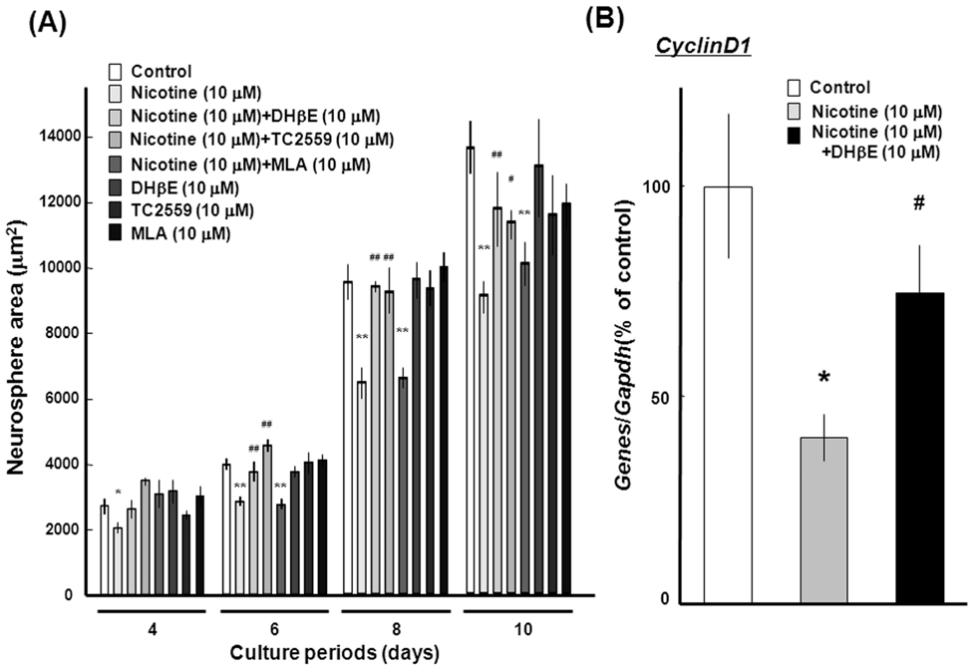
Effects of nAChR antagonists in undifferentiated mouse neural progenitors. (A) The selective nAChR antagonists DHβE, TC2559 and MLA were included in culture medium for determination of the total size of neurospheres during culture. (B) Cells were cultured with EGF in either the presence or absence of 10 µM nicotine and 10 µM DHβE for 10 days, followed by extraction of total RNA and subsequent RT-PCR analysis on *cyclinD1* gene. *P<0.05, **P<0.01, significantly different from each control value obtained in cells not exposed to nicotine. ^#^P<0.05, ^##^P<0.01, significantly different from the value obtained in cells exposed to nicotine alone.

The decrease by 10 µM nicotine was significantly prevented by simultaneous exposure to the heteromeric α4β2 nAChR subtype antagonists DHβE at 10 µM and TC2559 at 10 µM, but not by the homomeric α7 nAChR subtype antagonist MLA at 10 µM, with regards to the total area of neurospheres formed with clustered proliferating cells during culture for 10 days (Fig. 7A). To investigate the mechanism underlying the nicotine-induced decrease in proliferation, mRNA expression was assessed for cyclinD1, which is a key regulator of cell cycle. For instance, proneural neurogenin-2 induces cell cycle arrest during neurogenesis in association with rapidly decreased expression of a subset of the cell cycle regulators, cyclins [29]. Marked expression was seen for *cyclinD1* mRNA in neurospheres cultured for 10 days, while sustained exposure to nicotine at 10 µM led to a profound decrease in *cyclinD1* mRNA expression in a manner sensitive to the prevention by DHβE at 10 µM (Fig. 7B).

**Figure 8:**
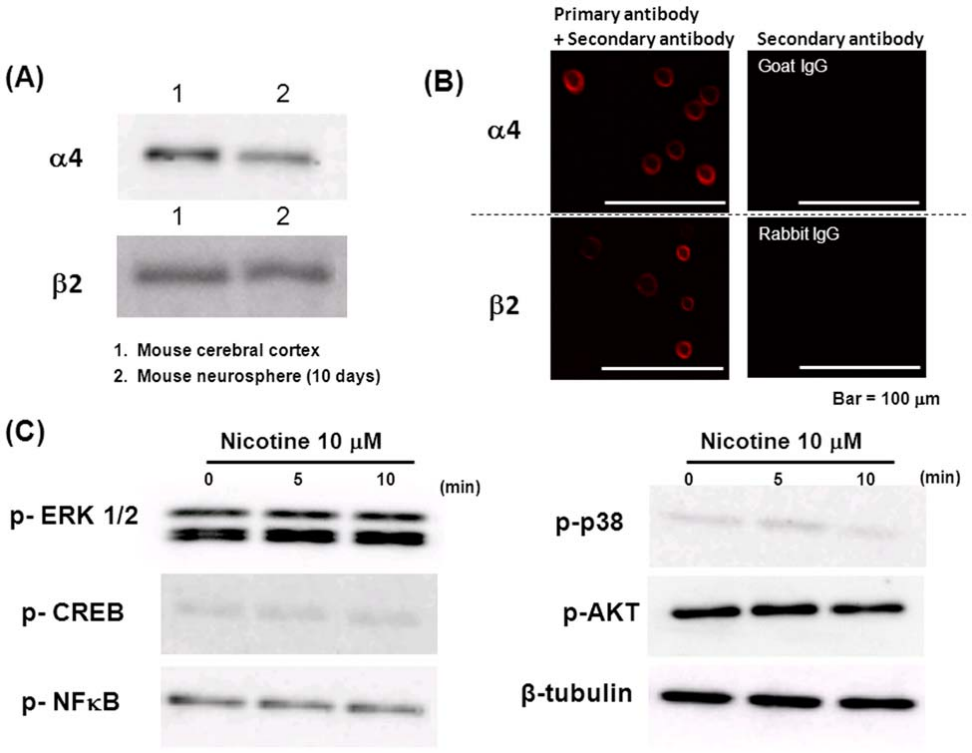
Expression and phosphorylation of particular proteins in undifferentiated mouse progenitors exposed to nicotine. Undifferentiated mouse progenitors were cultured with EGF for 10 days and subjected to (A) Western blotting and (B) immunocytochemistry for α4 and β2 subunits of nAChRs. Immunocytochemical images are also shown with cells not treated with the primary antibody. (C) Undifferentiated cells were also exposed to 10 µM nicotine for 5 to 10 min for subsequent Western blotting. Typical pictures are shown with similar results in three independent sets of experiments.

Protein expression was seen for α4 and β2 subunits in undifferentiated neural progenitor cells cultured for 10 days at the corresponding molecular weight position identical to that of mouse whole brain on the gel by Western blotting (Fig. 8A). Similarly, marked immunoreactive proteins were detected for α4 and β2 subunits at the location with cell surface features in undifferentiated progenitors cultured for 10 days on immunocytochemical analysis (Fig. 8B, left panels), whereas no immunoreactivity was detected in cultured neural progenitors not treated with those primary antibodies (Fig. 8B, right panels). In these undifferentiated progenitors, however, nicotine failed to markedly affect the phosphorylation of particular kinases required for intracellular signaling. These included extracellular regulated MAP kinase (ERK) 1/2, cAMP responsive element binding protein (CREB), nuclear factor κB (NF-κB), p38 kinase (p38) and serine/threonine protein kinase Akt (AKT) (Fig. 8C).

### Differentiation of mouse neurospheres

**Figure 9:**
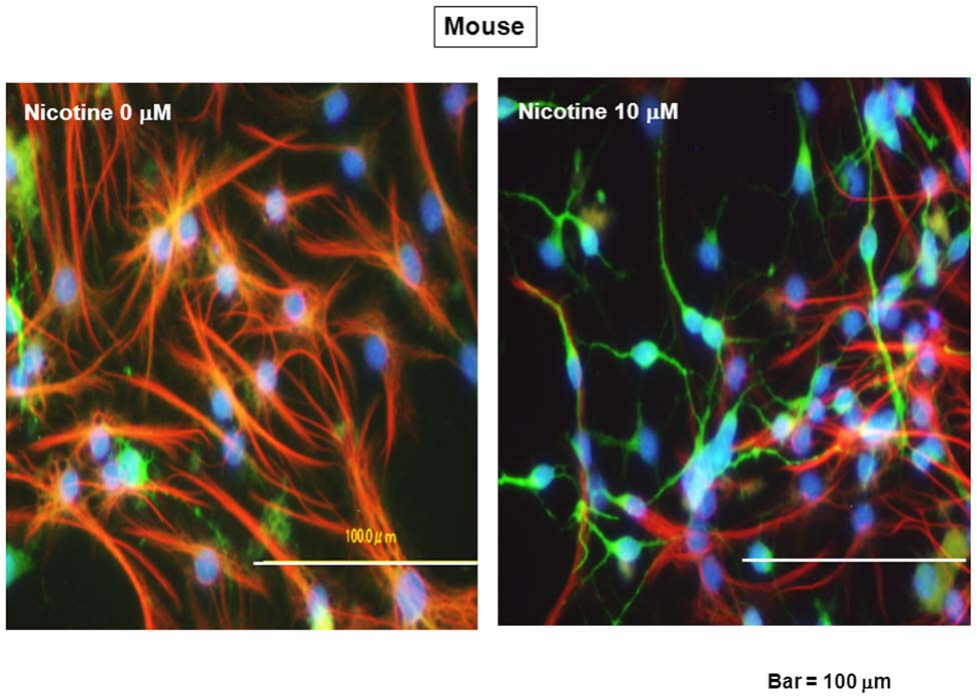
Differentiation of mouse progenitors. Cells were dispersed after the culture with EGF in either the presence or absence of 10 µM nicotine for 10 days, followed by further culture for an additional 4 days. Cells were then fixed for double immunocytochemical detection of both MAP2 and GFAP together with Hoechst33342 staining.

**Figure 10:**
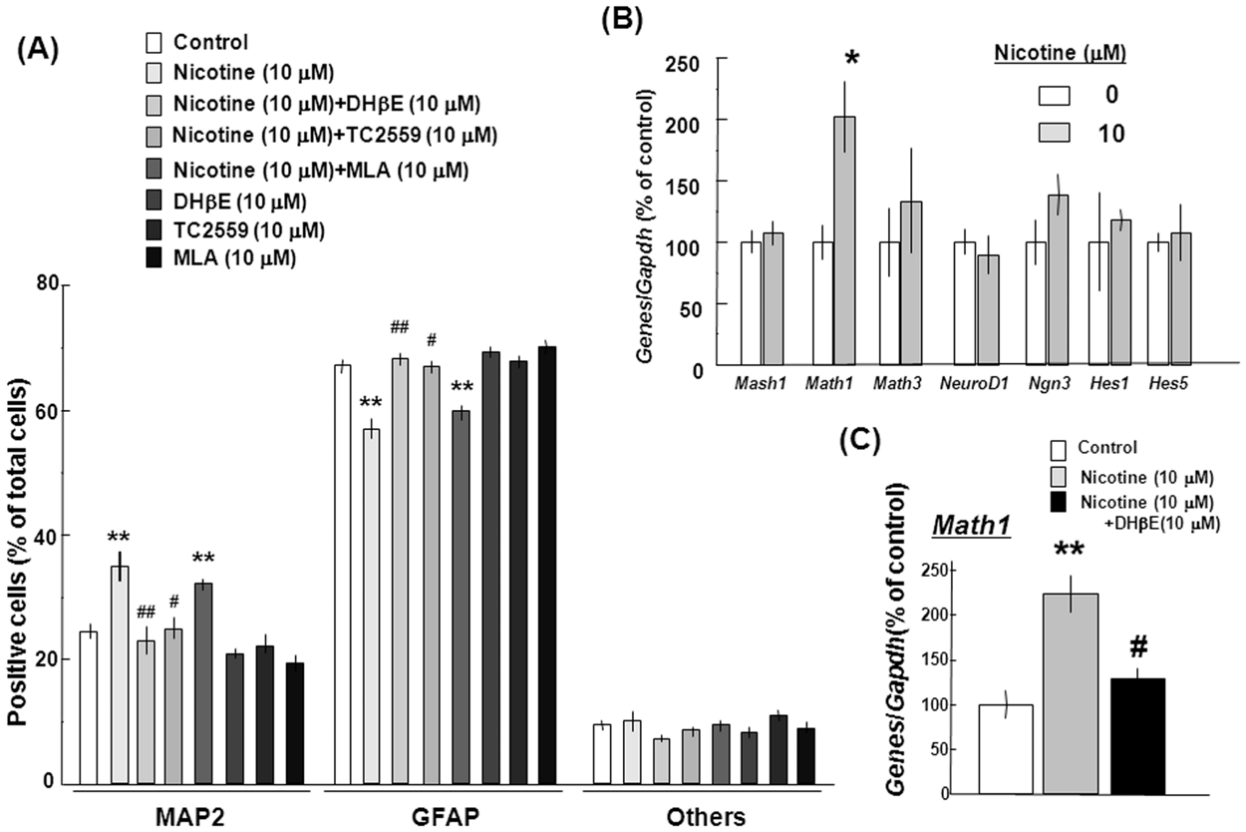
Effects of nicotine on differentiation of mouse progenitors. (A) Cells were cultured with EGF in either the presence or absence of 10 µM nicotine, 10 µM DHβE, 10 µM TC2559 and 10 µM MLA for 10 days, followed by dispersion after removal of EGF and subsequent double immunocytochemistry analysis along with Hechst33342 staining for counting the number of immunoreactive cells. (B) Cells were cultured with EGF for 10 days in either the presence or absence of 10 µM nicotine, followed by extraction of total RNA and subsequent RT-PCR analysis on different bHLH genes. (C) Cells were cultured with EGF in either the presence or absence of 10 µM nicotine and 10 µM DHβE, followed by extraction of total RNA and subsequent RT-PCR analysis on *Math1* gene. *P<0.05, **P<0.01, significantly different from each control value obtained in cells not exposed to nicotine. ^##^P<0.01, significantly different from the control value obtained in cells exposed to nicotine alone.

Murine neurospheres were cultured in either the presence or absence of nicotine at 10 µM for 10 days in the presence of EGF, followed by dispersion after the removal of EGF and nicotine to initiate spontaneous differentiation and subsequent further culture for an additional 4 days toward double immunocytochemistry analysis on the neuronal marker MAP2 and astroglial marker GFAP. Double immunocytochemistry analysis clearly revealed the presence of numbers of cells immunoreactive for either MAP2 or GFAP amongst cultured cells stained with Hoechst33342 when determined 4 days after dispersion (Fig. 9). For quantitative analysis, the number of cells immunoreactive for either MAP2 or GFAP was individually counted for calculation of the percentage over the total number of cells stained with Hoechst33342. Around 70% of cells were immunoreactive for GFAP and almost 20% for MAP2 after spontaneous differentiation (Fig. 10A). Prior exposure to nicotine alone significantly increased the number of cells immunoreactive for MAP2 with a significant decrease in that for GFAP, while these changes were significantly prevented by simultaneous exposure to 10 µM DHβE and 10 µM TC2559, but not by that to 10 µM MLA.

An attempt was next made to determine whether sustained exposure to nicotine leads to the altered expression profile of a variety of basic helix-loop-helix (bHLH) genes responsible for the positive and negative regulation of neuronal differentiation of undifferentiated neural stem cells [30]. These included the activator (Mash1, Math1, Math3, NeuroD1 and Ngn3) and repressor (Hes1 and Hes5) types of bHLH factors. In undifferentiated murine progenitors cultured with nicotine at 10 µM for 10 days, significant and selective upregulation was seen in mRNA expression of the activator-type *Math1*, but not in that of other activator- and repressor-type factors tested (Fig. 10B). Upregulation of *Math1* mRNA expression was again sensitive to the prevention by the addition of 10 µM DHβE (Fig. 10C).

### Progenitors from neocortex of embryonic NMDAR1-null mice

**Figure 11:**
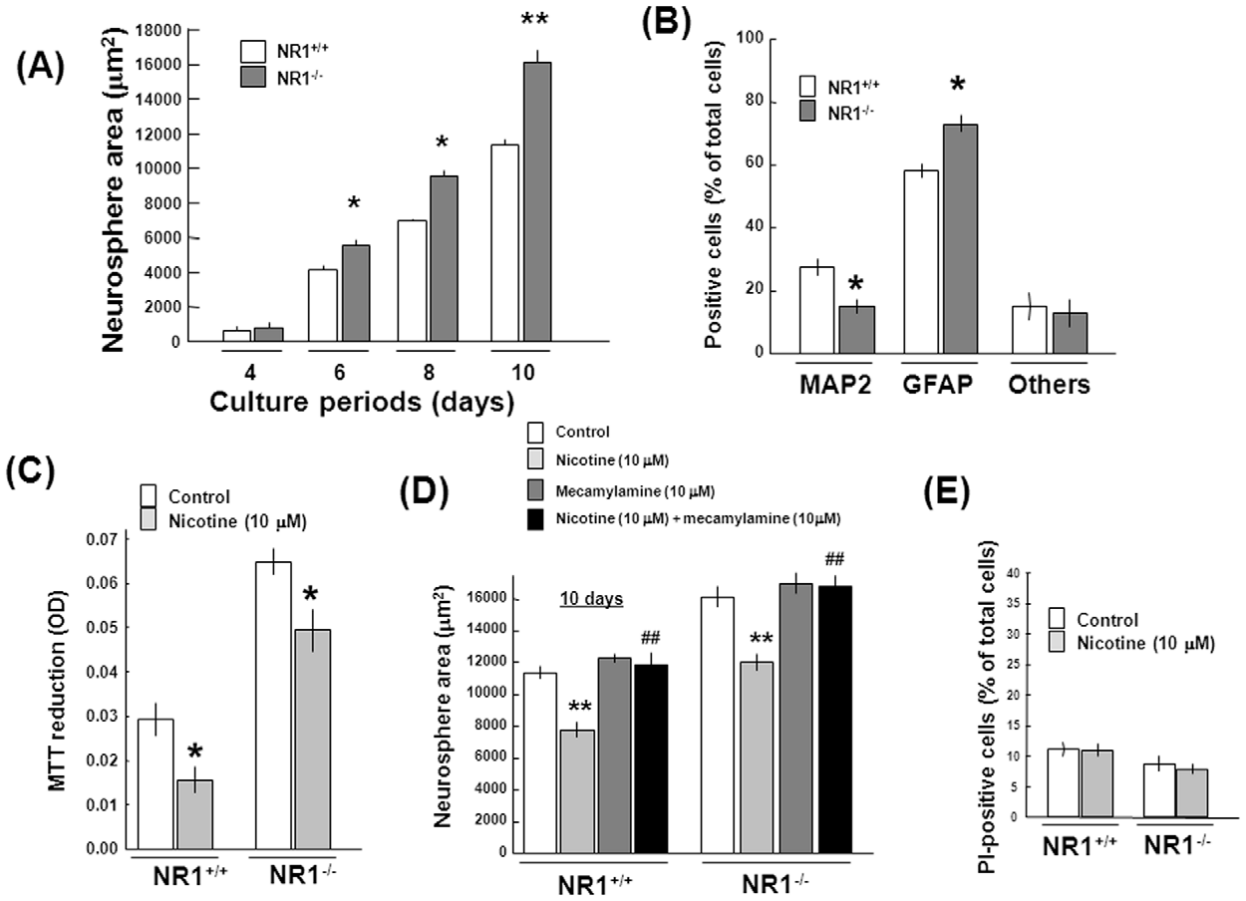
Neural progenitors prepared from NMDAR1-null mice. Neocortex was dissected from WT (NR1^+/+^) and NMDAR1-null (NR1^−/−^) mice, followed by culture with EGF for 10 days and subsequent determination of (A) the total size of neurospheres and (B) the number of individual immunoreactive cells on double immunocytochemistry analysis along with Hechst33342 staining. *P<0.05, **P<0.01, significantly different from the value obtained cells from WT mice. Cells from WT and NMDAR1-null mice were also cultured with EGF in either the presence or absence of 10 µM nicotine, 10 µM DHβE and 10 µM MLA for 10 days, followed by determination of (C) MTT reduction, (D) neurosphere size and (E) PI-positive dead cells. *P<0.05, **P<0.01, significantly different from each control value obtained in cells not exposed to nicotine. ^##^P<0.01, significantly different from the control value obtained in cells exposed to nicotine alone.

As similar pharmacological profiles are shown in undifferentiated rat progenitors exposed to NMDA [31], progenitors were isolated from neocortex of embryonic mice deficient of NMDAR1 subunit (NR1^−/−^) to evaluate the possible common mechanism required for regulation of their proliferation and differentiation by signal inputs between ionotropic NMDAR and nAChR channels. The total size was invariably larger in neurospheres formed by clustered proliferating cells from NMDAR1-null mice than in those from wild-type (WT; NR1^+/+^) mice during the culture from 6 to 10 days (Fig. 11A). In NMDAR1-null cells cultured for 4 days after removal of EGF, a significant decrease was found in the number of MAP2-positive cells with increased GFAP-positive cells compared with cells from WT mice (Fig. 11B). However, similarly significant inhibition was seen in MTT reduction of undifferentiated progenitors isolated from WT and NMDAR1-null mice after sustained exposure to nicotine at 10 µM for 10 days (Fig. 11C). In both WT and NMDAR1-null progenitors, the total size of neurospheres was also significantly decreased following sustained exposure to 10 µM nicotine for 10 days in a manner prevented by the general nAChR antagonist mecamylamine (Fig. 11D). In any situations employed, moreover, no significant change was induced in the ratio of PI-positive cells over Hoechst33342-positive cells irrespective of the animal genotype (Fig. 11E).

## Discussion

The essential importance of the present findings is that sustained exposure to nicotine led to a significant decrease in the capability to form neurospheres by clusters of proliferating cells along with subsequent promotion of the ability to express the neuronal marker MAP2 in a manner sensitive to the heteromeric α4β2 subtype antagonists DHβE and TC2559 in cultured neural progenitor cells isolated from fetal mouse neocortex. These findings, coupled with the present data from RT-PCR, Western blotting and immunocytochemical analyses, undoubtedly give rise to an idea that functional heteromeric α4β2 nAChR channels would be indeed expressed to play a pivotal dual role in the proliferation toward self-replication and the commitment/differentiation into particular progeny lineages in neural progenitors during brain development. Evidence is accumulating for the functional expression of nAChRs by undifferentiated neural progenitor cells before commitment and differentiation. In progenitors from the fetal mouse cortex, nAChR agonists such as acetylcholine and nicotine evoke intracellular Ca^2+^ signals in a manner sensitive to the prevention by DHβE [32]. In human neural progenitor cells differentiated from human embryonic stem cells, marked mRNA is seen for a variety of ionotropic subtypes of glutamatergic, GABAergic, nicotinic and purinergic receptors, in addition to different subunits of voltage-sensitive Ca^2+^- and Na^+^-channels after removal of bFGF [33]. In the murine hippocampal DG, α7 subunit is found to be essential for normal survival, maturation and integration of adult-born neurons [34].

Although a report is available for the role of nAChR in proliferation and differentiation of the pluripotent P19 cells derived from murine embryonal carcinoma in the literature as well [35], this is the first direct demonstration of functional expression of an nAChR subtype composed of α4β2 subunits endowed to positively regulate neuronal differentiation in cultured neural progenitor cells from fetal mouse neocortex. Deteriorated astroglial differentiation would be brought about as a consequence of promoted neuronal differentiation from neural progenitors. In our previous studies [36], moreover, sustained activation of ionotropic GABA_A_ receptor and NMDAR regulates the proliferation to an opposite direction, in addition to differentially modulating spontaneous and induced differentiation into neurons, in neural progenitors isolated from fetal rodent brains. In particular, activation of NMDAR leads to the inhibition of proliferation with concomitant promotion of subsequent neuronal differentiation in neural progenitors isolated from fetal rat brains [31] as shown with nAChR in this study. Accordingly, neurogenesis could be also under the delicate control by ionotropic signal inputs mediated by GABAergic, glutamatergic and nicotinic cholinergic machineries in developing brains as seen with the neurotransmission in adult brains. By taking into consideration the fact that nicotine was still effective in inhibiting the proliferation activity of neural progenitors from NR1^−/−^ mice, activation of nAChR would likely result in negative regulation of self-replication along with promotion of subsequent neuronal differentiation through a mechanism irrelevant to NMDAR signal inputs in undifferentiated neural progenitors.

The present pharmacological profiling argues in favor of an idea that nicotine would be endowed to at least in part modulate the cellular proliferation and differentiation of neural progenitor cells through a mechanism relevant to the influx of extracellular Ca^2+^ ions across cell membranes after activation of heteromeric α4β2 nAChR channels permeable for Ca^2+^ ions. The data presented here are in a good agreement with previous findings on the differentiation fate of neural progenitors toward particular progeny lineages. Activation of either NMDAR-operated or voltage-sensitive L-type Ca^2+^ channels leads to the inhibition of expression of the glial fate genes Hes1 and Id2, with concomitant promotion of expression of the positive regulator of neuronal differentiation NeuroD, in adult rat hippocampal neural progenitor cells [37]. Similarly, we have demonstrated that prior activation of NMDAR results in the promotion of subsequent differentiation into cells immunoreactive for MAP2, neuron-specific enolase and neuronal nuclei in adult and embryo murine neural progenitor cells [14]. In embryonic rat hippocampal neural progenitor cells repetitively exposed to NMDA, furthermore, marked facilitation is seen with the acquisition of a neuronal phenotype accompanied by the increased expression of pro-neuronal bHLH factors [38]. The present findings that selective upregulation of *Math1* was seen in undifferentiated neural progenitors exposed to nicotine at a relatively high concentration give support to the possible involvement of Math1 in mechanisms underlying the promoted neuronal differentiation mediated by activation of nAChR. In fact, Math1 is a mouse homologue of the Drosophila gene atonal, and encodes a bHLH transcription factor that is essential for neurogenesis [39], [40]. No direct evidence for the involvement of intracellular free Ca^2+^ levels in Math1 expression is available in the literature to date, whereas the data cited above are all suggestive of the proposal that α4β2 nAChR signals would promote the commitment/differentiation into neurons through a mechanism relevant to the transactivation of Math1 gene in association with deterioration of the self-renewal capacity in neural progenitor cells during brain development. However, the final conclusion should await the evaluation using knockdown and overexpression strategies on α4β2 nAChR subtype in neural progenitors *in vitro* and animals *in vivo*.

In a previous case-controlled study, an inverse correlation is seen between smoking and Alzheimer's disease incidence [41]. In line with these findings on Alzheimer's disease, epidemiological reports are available for the decreased incidence of Parkinson's disease in smokers without any correlation to mortality in the literature [42], [43]. However, the present requirement of relatively high concentrations of nicotine for the promotion of neuronal differentiation from progenitors is unfavorable for an idea that smoking would accelerate the neuronal differentiation in the brains of smokers. By contrast, an agonist at α4β2 nAChR subtype would be beneficial for the recovery and/or rehabilitation through a mechanism related to the promoted neuronal differentiation from endogenous neural progenitor cells expressed in particular structures of adult matured brains toward the prevention of consequential neuronal loss progression in patients suffering from a variety of neurodegenerative diseases in a particular situation. Although this study was originally aimed at evaluating pharmacological properties of the cognitive enhancer nicotine in neural progenitors, the possibility that the present findings give us a clue to elucidate a novel property for particular nAChR subtypes expressed in the brain to regulate embryonic and adult neurogenesis is not ruled out.

## Conclusion

It thus appears that functional α4β2 nAChR subtype is constitutively expressed in undifferentiated neural progenitor cells before commitment for the regulation of cellular proliferation toward self-replication and subsequent differentiation into particular lineages in fetal rodent neocortex. Modulation of the functionality of α4β2 nAChR subtype could be thus of a great benefit for the regeneration and/or supplementation without surgical implantations of progenitor cells in patients suffering from a variety of brain diseases pertaining to neuronal and/or astroglial dysfunctions. Alternatively, prior modulation of the activity of the α4β2 nAchR subtype could be a novel strategy for subsequent regulation of differentiation toward a neuronal lineage of neural progenitors implanted.
